# Persistent shorter and longer sleep duration from infancy to childhood and its prospective association with chronic depression symptoms

**DOI:** 10.1192/j.eurpsy.2025.504

**Published:** 2025-08-26

**Authors:** R. L. M. Amos, B. B. Durdurak, A. M. Gregory, S. Marwaha, I. Morales-Muñoz

**Affiliations:** 1 Research & Development, Birmimgham and Solihull Mental Health Foundation Trust; 2 School of Psychology, Institute for Mental Health, University of Birmingham, Birmingham; 3 Department of Psychology, Royal Holloway University of London, London, United Kingdom

## Abstract

**Introduction:**

Identifying early-life risk factors for chronic depression symptomology in young people, is essential to informing early targeted interventions. One highly prevalent symptom (and potential risk factor) in depression is sleep problems, such as insomnia or hypersomnia. However, most studies have measured sleep disturbances and depression symptoms at only one time point, and the prospective relationship between *persistent* shorter or longer sleep duration in childhood and *chronic depression symptoms* in adolescence through to adulthood has not been explored.

**Objectives:**

To identify whether longitudinal trajectories of persistent shorter sleep and persistent longer sleep duration between 6 months to 7 years of age, are associated with increased risk of developing chronic depression symptoms between 13-22 years of age.

**Methods:**

Prospective associations were explored using the Avon Longitudinal Study of Parents and Children (ALSPAC), in the UK. Childhood night-time sleep duration was parent-reported at 6, 18, and 30 months and at 3.5, 4 to 5, 5 to 6, and 6 to 7 years. Depression symptoms were self-reported via the Short Mood and Feelings Questionnaire (SMFQ) at, 12.5, 13.5, 16, 17.5, 18, 21 and 22 years of age. Latent Growth Curve Analysis was used to identify longitudinal trajectories of night-time sleep duration from 6 months to 7 years of age (i.e. longer (63%), shorter (2%), average-shorter sleep (22%) and average-longer sleep (13%)) and depression symptoms (i.e. chronic (5%), non-chronic (95%)) from 13 to 22 years. Logistic regressions were conducted to identify the prospective association between persistent shorter and persistent longer sleep trajectories and chronic depression symptoms.

**Results:**

Preliminary results revealed that persistent shorter sleep duration across childhood was associated with increased likelihood of presenting with chronic depression symptoms, even after adjusting for the effects of sex, birthweight, maternal age, child ethnicity, family adversity and maternal socioeconomic status (OR = 1.94, 95% CIs, 1.01, 3.73 p =. 046). Persistent longer sleep however did not show significant associations.

**Conclusions:**

A persistent pattern of shorter sleep duration across childhood is associated with chronic depression symptoms in adolescence through to adulthood. Sleep is a modifiable risk factor and targeted interventions for those presenting a sustained pattern of shorter sleep duration across childhood is suggested to prevent future mental health problems, such as depression.

**Disclosure of Interest:**

None Declared

